# 2-(2-Chloro-8-methyl­quinolin-3-yl)-4-phenyl-1,2-di­hydro­quinazoline

**DOI:** 10.1107/S1600536813029334

**Published:** 2013-10-31

**Authors:** Chamseddine Derabli, Raouf Boulcina, Sofiane Bouacida, Hocine Merazig, Abdelmadjid Debache

**Affiliations:** a Laboratoire de Synthèse des Molécules d’intérêts Biologiques, Département de Chimie, Faculté des Sciences Exactes, Université de Constantine 1, 25000 Constantine, Algeria; b Unité de Recherche de Chimie de l’Environnement et Moléculaire Structurale, CHEMS, Université Constantine 1, 25000, Algeria; c Département Sciences de la Matière, Faculté des Sciences Exactes et Sciences de la Nature et de la Vie, Université Oum El Bouaghi 04000, Algeria

## Abstract

In the title compound, C_24_H_18_ClN_3_, the di­hydro­quinazoline and methyl-substituted quinoline benzene rings make a dihedral angle of 78.18 (4)° and form dihedral angles of 45.91 (5) and 79.80 (4)°, respectively, with the phenyl ring. The dihedral angle between the phenyl ring of di­hydro­quinazoline and the methyl-substituted benzene ring of quinoline is 78.18 (4)°. The crystal packing can be described as crossed layers parallel to the (011) and (0-11) planes. The structure features N—H⋯N hydrogen bonds and π–π inter­actions [centroid–centroid distance between phenyl rings = 3.7301 (9) Å].

## Related literature

For the preparation and applications of quinazoline and quinoline derivatives, see: Jenekhe *et al.* (2001 ▶); Hoemann *et al.* (2000 ▶); Connolly *et al.* (2005 ▶); Besson *et al.* (2007 ▶); Roma *et al.* (2000 ▶); Chen *et al.* (2001 ▶); Debache *et al.* (2008 ▶, 2009 ▶); Nemouchi *et al.* (2012 ▶).
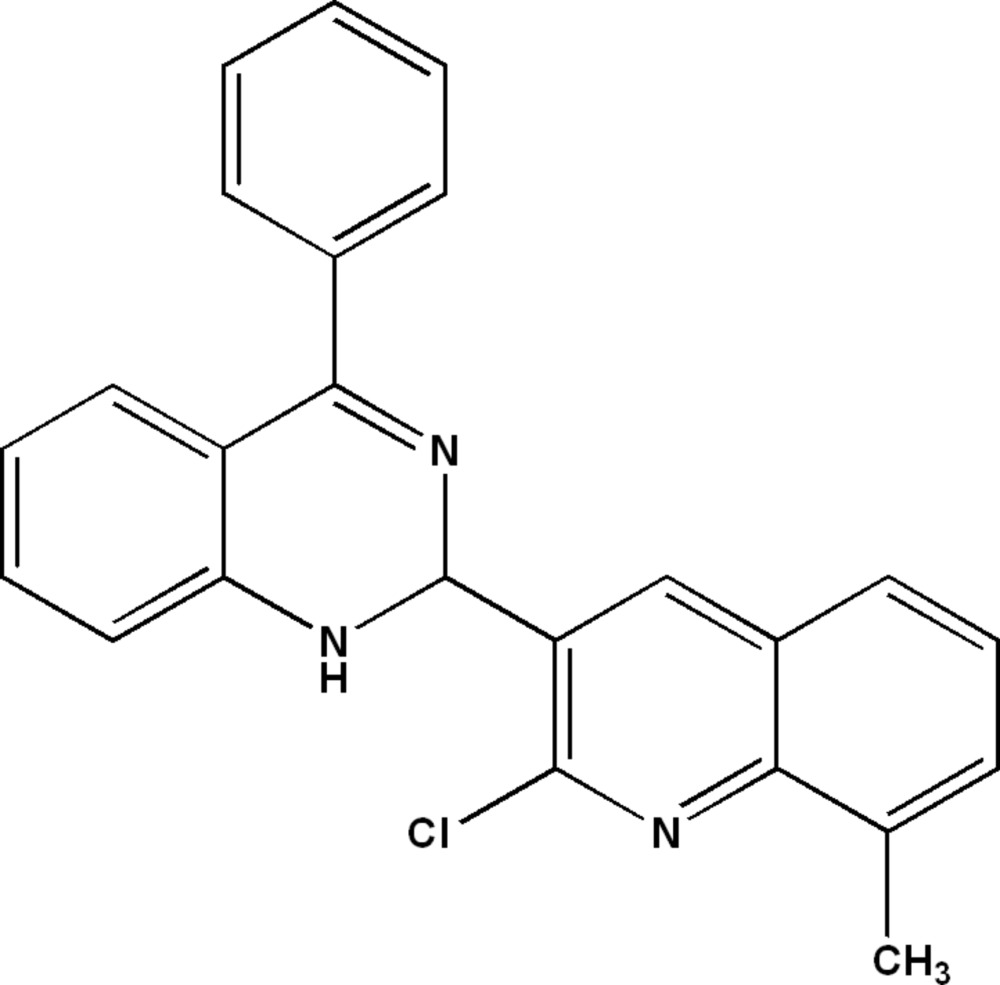


## Experimental

### 

#### Crystal data

C_24_H_18_ClN_3_
*M* *_r_* = 383.86
Monoclinic, 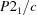

*a* = 14.4553 (13) Å
*b* = 8.7501 (9) Å
*c* = 16.8630 (16) Å
β = 119.696 (6)°
*V* = 1852.8 (3) Å^3^
*Z* = 4
Mo *K*α radiation
μ = 0.22 mm^−1^
*T* = 150 K
0.12 × 0.04 × 0.02 mm

#### Data collection

Bruker APEXII CCD area-detector diffractometer
Absorption correction: multi-scan (*SADABS*; Sheldrick, 2002 ▶) *T*_min_ = 0.959, *T*_max_ = 1.000
10282 measured reflections
3276 independent reflections
2894 reflections with *I* > 2σ(*I*)
*R*_int_ = 0.027

#### Refinement

*R*[*F* ^2^ > 2σ(*F* ^2^)] = 0.031
*wR*(*F* ^2^) = 0.082
*S* = 1.06
3276 reflections
254 parameters
H-atom parameters constrained
Δρ_max_ = 0.26 e Å^−3^
Δρ_min_ = −0.26 e Å^−3^

### 

Data collection: *APEX2* (Bruker, 2011 ▶); cell refinement: *SAINT* (Bruker, 2011 ▶); data reduction: *SAINT*; program(s) used to solve structure: *SIR2002* (Burla *et al.*, 2003 ▶); program(s) used to refine structure: *SHELXL97* (Sheldrick, 2008 ▶); molecular graphics: *ORTEP-3 for Windows* (Farrugia, 2012 ▶) and *DIAMOND* (Brandenburg & Berndt, 2001 ▶); software used to prepare material for publication: *WinGX* (Farrugia, 2012 ▶).

## Supplementary Material

Crystal structure: contains datablock(s) I. DOI: 10.1107/S1600536813029334/hg5353sup1.cif

Structure factors: contains datablock(s) I. DOI: 10.1107/S1600536813029334/hg5353Isup2.hkl

Supplementary material file. DOI: 10.1107/S1600536813029334/hg5353Isup3.cml

Additional supplementary materials:  crystallographic information; 3D view; checkCIF report

## Figures and Tables

**Table 1:** Hydrogen-bond geometry (Å, °)

*D*—H⋯*A*	*D*—H	H⋯*A*	*D*⋯*A*	*D*—H⋯*A*
N3—H3*N*⋯N2^i^	0.86	2.26	3.0998 (18)	165

Symmetry code: (i) 
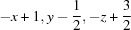
.

**Fig. 1.**
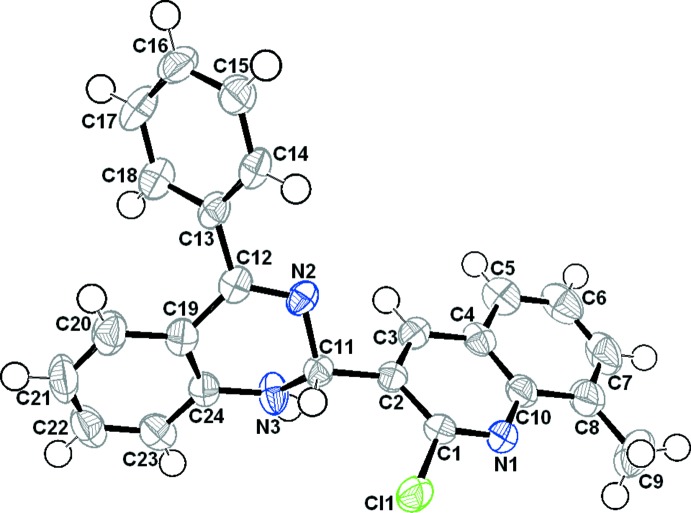
The title molecule (Farrugia, 2012) with the atomic labelling scheme. The displacement parameters are drawn at the 50% probability level.

**Fig. 2.**
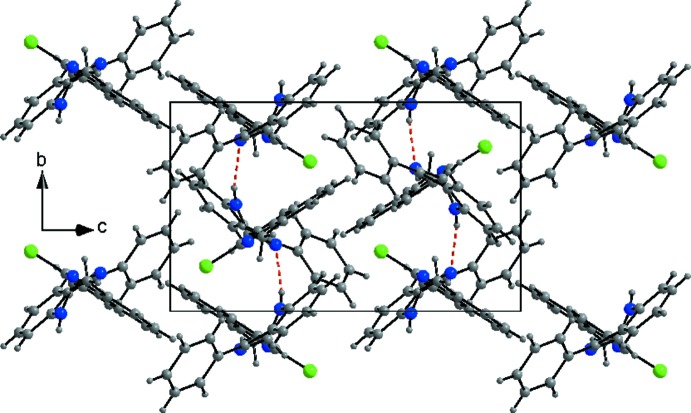
(Brandenburg & Berndt, 2001) Part of the crystal structure viewed down the b axis showing the hydrogen bonds N—H···N as dashed red lines.

## supplementary crystallographic information

### 1. Comment

It is well known that the quinoline ring system is an important structural unit widely existing in alkaloids, therapeutics and synthetic analogues with interesting biological activities (Roma *et al.*, 2000; Chen *et al.*, 2001). In addition quinolines are valuable synthons for the preparation of nano- and *meso*-structures with enhanced electronic and photonic functions (Jenekhe *et al.*, 2001). Due to their importance as substructures in a broad range of natural and designed products, significant efforts continue to be directed into the development of new quinoline-based structures (Hoemann *et al.*, 2000). In the other hand, the quinazoline unit represents a useful natural product scaffold with demonstrated activities against numerous disorders. The transferable nature of its properties provides a strong rationale for the development of synthetic methods. Not surprisingly, considerable progress in synthetic methodology applicable to quinazoline alkaloids has been made during the past decade (Connolly *et al.*, 2005; Besson *et al.*, 2007). As part of our research in developing new efficient methods for heterocycles synthesis and also multicomponent reactions (Debache *et al.*, 2008; Debache *et al.*, 2009; Nemouchi *et al.*, 2012), we decided to design some new molecules containing reactive fonctionnalities. As a result of this investigation we report herein a fast and efficient protocol for the synthesis of 2-(2-Chloro-8-methylquinolin-3-yl)-4-phenyl-1,2-dihydroquinazoline *via* a three-component reaction between 2-aminobenzophenone, 2-chloro-8-methylquinoline-3-carbaldehyde, and ammonium acetate, completed by the X-ray structure analysis.

The molecular geometry and the atom-numbering scheme of (I) are shown in Fig. 1. The benzene ring of dihydroquinazoline and methyl-substituted benzene rings of quinoline form a dihedral angles of 45.91 (5) and 79.80 (4)° respectively with a phenyl ring group. The dihedral angle between the phenyl ring of dihydroquinazoline and methyl-substituted benzene rings of quinoline is 78.18 (4) °. The crystal packing can be described as crossed layers parallel to the (011) and (0–11) planes. (Fig. 2). It is stabilized by a N—H···N hydrogen bond (Table 1, Fig. 2) and π-π interactions. however the centroid to centroid small distance between the phenyl rings is 3.7301 (9) Å. These interactions link the molecules within the layers and also link the layers together and reinforcing the cohesion of the structure.

### 2. Experimental

A mixture of 2-chloro-8-methylquinoline-3-carbaldehyde (1.0 equiv), 2-aminobenzophenone (1.0 equiv), ammonium acetate (2.0 equiv), and 4-(*N*,*N*-dimethylamino)pyridine (0.2 equiv.) in 5 ml of absolute ethanol was stirred at 40°C. After completion of the reaction as monitored by TLC, the reaction mixture was poured into ice cold water; solid product was filtered, washed with water (3–5 ml) and dried. The crude product was recrystallized from ethyl acetate to give pure dihydroquinazoline as a yellow solid; m.p. 182–184 °C; IR (KBr) ν 3329, 1605, 1551, 1470, 1315, 1080, 756, 698 cm-1; 1H NMR (CDCl3, 250 MHz) δ 8.52 (s, 1H, arom.), 7.74–7.41 (m, 8H, arom.), 7.34–7.26 (m, 2H, arom.), 6.83–6.74 (m, 2H, arom.), 6.48 (s, 1H, CH), 4.79 (s, 1H, NH), 2.81 (s, 3H, CH3); 13 C NMR (CDCl3, 62.5 MHz) δ 167.4, 148.2, 146.8, 146.6, 139.3, 138.0, 136.4, 133.2, 132.6, 130.9, 129.9, 129.3, 123.0, 128.4, 127.5, 127.1, 126.1, 120.4, 118.9, 117.9, 114.8, 68.8, 18.0. Anal. calcd for C_24_H_18_N_3_Cl: C, 75.09; H, 4.73; N, 10.95; Found: C, 75.18; H, 4.94; N, 11.37. HRMS calcd f or C_24_H_19_N_3_Cl (MH+) 384.1189; found 384.1162.

### 3. Refinement

Hydrogen atoms were localized on Fourier maps but introduced in calculated positions and treated as riding on their parent atoms (C and N) with C—H = 0.96 Å (methyl); C—H = 0.93 Å (aromatic) or C—H = 0.98 Å (methine); N—H = 0.86 Å and with *U*_iso_(H) = 1.2 *U*_eq_(C_aryl_; C_methine_ or N) and *U*_iso_(H) = 1.5 *U*_eq_(C_methyl_).

### Crystal data

**Table:**

C_24_H_18_ClN_3_	*F*(000) = 800
*M**_r_* = 383.86	*D*_x_ = 1.376 Mg m^−^^3^
Monoclinic, *P*2_1_/*c*	Mo *K*α radiation, λ = 0.71073 Å
Hall symbol: -P 2ybc	Cell parameters from 4868 reflections
*a* = 14.4553 (13) Å	θ = 2.4–25.1°
*b* = 8.7501 (9) Å	µ = 0.22 mm^−^^1^
*c* = 16.8630 (16) Å	*T* = 150 K
β = 119.696 (6)°	Stick, colourless
*V* = 1852.8 (3) Å^3^	0.12 × 0.04 × 0.02 mm
*Z* = 4	

### Data collection

**Table:**

Bruker APEXII CCD area-detector diffractometer	2894 reflections with *I* > 2σ(*I*)
Graphite monochromator	*R*_int_ = 0.027
φ and ω scans	θ_max_ = 25.1°, θ_min_ = 2.7°
Absorption correction: multi-scan (*SADABS*; Sheldrick, 2002)	*h* = −17→17
*T*_min_ = 0.959, *T*_max_ = 1.000	*k* = −10→10
10282 measured reflections	*l* = −20→20
3276 independent reflections	

### Refinement

**Table:**

Refinement on *F*^2^	Primary atom site location: structure-invariant direct methods
Least-squares matrix: full	Secondary atom site location: difference Fourier map
*R*[*F*^2^ > 2σ(*F*^2^)] = 0.031	Hydrogen site location: inferred from neighbouring sites
*wR*(*F*^2^) = 0.082	H-atom parameters constrained
*S* = 1.06	*w* = 1/[σ^2^(*F*_o_^2^) + (0.0364*P*)^2^ + 0.8263*P*] where *P* = (*F*_o_^2^ + 2*F*_c_^2^)/3
3276 reflections	(Δ/σ)_max_ = 0.001
254 parameters	Δρ_max_ = 0.26 e Å^−^^3^
0 restraints	Δρ_min_ = −0.26 e Å^−^^3^

### Special details

**Table:**

Geometry. All e.s.d.'s (except the e.s.d. in the dihedral angle between two l.s. planes) are estimated using the full covariance matrix. The cell e.s.d.'s are taken into account individually in the estimation of e.s.d.'s in distances, angles and torsion angles; correlations between e.s.d.'s in cell parameters are only used when they are defined by crystal symmetry. An approximate (isotropic) treatment of cell e.s.d.'s is used for estimating e.s.d.'s involving l.s. planes.
Refinement. Refinement of *F*^2^ against ALL reflections. The weighted *R*-factor *wR* and goodness of fit *S* are based on *F*^2^, conventional *R*-factors *R* are based on *F*, with *F* set to zero for negative *F*^2^. The threshold expression of *F*^2^ > σ(*F*^2^) is used only for calculating *R*-factors(gt) *etc*. and is not relevant to the choice of reflections for refinement. *R*-factors based on *F*^2^ are statistically about twice as large as those based on *F*, and *R*- factors based on ALL data will be even larger.

### Fractional atomic coordinates and isotropic or equivalent isotropic displacement parameters (Å^2^)

**Table:**

	*x*	*y*	*z*	*U*_iso_*/*U*_eq_	
C1	0.69552 (11)	0.67980 (16)	0.79870 (9)	0.0147 (3)	
C2	0.59188 (11)	0.61713 (15)	0.74927 (9)	0.0127 (3)	
C3	0.57150 (11)	0.53348 (16)	0.67390 (9)	0.0146 (3)	
H3	0.5049	0.4887	0.6391	0.018*	
C4	0.64981 (11)	0.51388 (16)	0.64785 (9)	0.0159 (3)	
C5	0.63045 (12)	0.43142 (17)	0.56897 (10)	0.0209 (3)	
H5	0.5646	0.3853	0.5328	0.025*	
C6	0.70818 (13)	0.41953 (19)	0.54594 (11)	0.0254 (4)	
H6	0.6951	0.3665	0.4936	0.031*	
C7	0.80782 (13)	0.4873 (2)	0.60120 (11)	0.0282 (4)	
H7	0.8602	0.4765	0.5849	0.034*	
C8	0.83123 (12)	0.5691 (2)	0.67858 (11)	0.0269 (4)	
C9	0.93732 (14)	0.6438 (3)	0.73654 (13)	0.0489 (6)	
H9A	0.9794	0.6349	0.7071	0.073*	
H9B	0.9734	0.5944	0.795	0.073*	
H9C	0.927	0.7498	0.7446	0.073*	
C10	0.74979 (11)	0.58353 (17)	0.70258 (10)	0.0178 (3)	
C11	0.50792 (11)	0.63352 (16)	0.77731 (9)	0.0131 (3)	
H11	0.5314	0.7111	0.8254	0.016*	
C12	0.32475 (11)	0.66315 (15)	0.71152 (9)	0.0138 (3)	
C13	0.21909 (11)	0.71284 (16)	0.63629 (10)	0.0146 (3)	
C14	0.21119 (11)	0.84104 (17)	0.58439 (10)	0.0167 (3)	
H14	0.2721	0.8973	0.5986	0.02*	
C15	0.11447 (11)	0.88597 (18)	0.51219 (10)	0.0202 (3)	
H15	0.1106	0.9722	0.4785	0.024*	
C16	0.02306 (12)	0.80291 (18)	0.48979 (10)	0.0217 (3)	
H16	−0.0421	0.8325	0.4409	0.026*	
C17	0.02994 (12)	0.67590 (18)	0.54081 (11)	0.0223 (3)	
H17	−0.0313	0.6204	0.5264	0.027*	
C18	0.12673 (11)	0.62970 (17)	0.61323 (10)	0.0193 (3)	
H18	0.1302	0.5431	0.6466	0.023*	
C19	0.33313 (11)	0.58635 (16)	0.79269 (10)	0.0158 (3)	
C20	0.25708 (12)	0.59518 (18)	0.82105 (10)	0.0215 (3)	
H20	0.2009	0.6641	0.7931	0.026*	
C21	0.26503 (13)	0.5023 (2)	0.89021 (11)	0.0261 (4)	
H21	0.2146	0.5094	0.909	0.031*	
C22	0.34775 (13)	0.39837 (19)	0.93189 (10)	0.0237 (4)	
H22	0.3507	0.333	0.9765	0.028*	
C23	0.42583 (12)	0.39113 (17)	0.90769 (10)	0.0192 (3)	
H23	0.482	0.3226	0.9367	0.023*	
C24	0.41994 (11)	0.48728 (16)	0.83933 (9)	0.0145 (3)	
N1	0.77105 (9)	0.66658 (15)	0.77866 (8)	0.0185 (3)	
N2	0.40582 (9)	0.68184 (13)	0.69974 (8)	0.0136 (3)	
N3	0.49626 (9)	0.48955 (14)	0.81327 (8)	0.0177 (3)	
H3N	0.5343	0.4108	0.818	0.021*	
Cl1	0.72959 (3)	0.78522 (4)	0.89821 (2)	0.02018 (12)	

### Atomic displacement parameters (Å^2^)

**Table:**

	*U*^11^	*U*^22^	*U*^33^	*U*^12^	*U*^13^	*U*^23^
C1	0.0142 (7)	0.0167 (7)	0.0124 (7)	0.0004 (6)	0.0059 (6)	0.0021 (6)
C2	0.0125 (7)	0.0110 (7)	0.0142 (7)	0.0021 (5)	0.0064 (6)	0.0036 (5)
C3	0.0134 (7)	0.0122 (7)	0.0170 (7)	−0.0001 (6)	0.0064 (6)	0.0020 (6)
C4	0.0191 (7)	0.0134 (7)	0.0168 (7)	0.0053 (6)	0.0100 (6)	0.0053 (6)
C5	0.0259 (8)	0.0171 (7)	0.0212 (8)	0.0033 (6)	0.0129 (7)	−0.0001 (6)
C6	0.0348 (9)	0.0253 (9)	0.0221 (8)	0.0093 (7)	0.0186 (7)	0.0019 (7)
C7	0.0273 (9)	0.0406 (10)	0.0267 (8)	0.0135 (8)	0.0209 (8)	0.0093 (8)
C8	0.0184 (8)	0.0441 (10)	0.0214 (8)	0.0068 (7)	0.0122 (7)	0.0075 (7)
C9	0.0188 (9)	0.1013 (19)	0.0341 (10)	−0.0071 (10)	0.0188 (8)	−0.0086 (11)
C10	0.0157 (7)	0.0236 (8)	0.0155 (7)	0.0050 (6)	0.0089 (6)	0.0058 (6)
C11	0.0124 (7)	0.0129 (7)	0.0138 (7)	−0.0008 (5)	0.0064 (6)	−0.0002 (6)
C12	0.0146 (7)	0.0101 (7)	0.0176 (7)	−0.0012 (5)	0.0088 (6)	−0.0041 (5)
C13	0.0124 (7)	0.0160 (7)	0.0173 (7)	0.0009 (6)	0.0087 (6)	−0.0039 (6)
C14	0.0129 (7)	0.0180 (7)	0.0215 (7)	−0.0008 (6)	0.0102 (6)	−0.0027 (6)
C15	0.0188 (7)	0.0232 (8)	0.0211 (7)	0.0056 (6)	0.0118 (6)	0.0041 (6)
C16	0.0125 (7)	0.0302 (9)	0.0201 (8)	0.0047 (6)	0.0064 (6)	−0.0008 (7)
C17	0.0122 (7)	0.0241 (8)	0.0299 (8)	−0.0023 (6)	0.0098 (7)	−0.0041 (7)
C18	0.0162 (7)	0.0179 (7)	0.0251 (8)	0.0001 (6)	0.0112 (6)	0.0003 (6)
C19	0.0166 (7)	0.0146 (7)	0.0179 (7)	−0.0029 (6)	0.0099 (6)	−0.0041 (6)
C20	0.0195 (8)	0.0251 (8)	0.0243 (8)	−0.0009 (6)	0.0142 (7)	−0.0052 (7)
C21	0.0265 (8)	0.0354 (10)	0.0268 (8)	−0.0073 (7)	0.0210 (7)	−0.0067 (7)
C22	0.0304 (9)	0.0258 (8)	0.0198 (8)	−0.0097 (7)	0.0160 (7)	−0.0023 (7)
C23	0.0229 (8)	0.0172 (7)	0.0176 (7)	−0.0027 (6)	0.0102 (6)	−0.0012 (6)
C24	0.0164 (7)	0.0139 (7)	0.0149 (7)	−0.0037 (6)	0.0090 (6)	−0.0045 (6)
N1	0.0126 (6)	0.0272 (7)	0.0156 (6)	0.0003 (5)	0.0071 (5)	0.0034 (5)
N2	0.0113 (6)	0.0122 (6)	0.0172 (6)	0.0008 (5)	0.0069 (5)	0.0003 (5)
N3	0.0198 (6)	0.0148 (6)	0.0255 (7)	0.0062 (5)	0.0166 (6)	0.0058 (5)
Cl1	0.01497 (19)	0.0289 (2)	0.01610 (19)	−0.00583 (15)	0.00722 (15)	−0.00646 (15)

### Geometric parameters (Å, º)

**Table:**

C1—N1	1.2981 (18)	C12—C19	1.475 (2)
C1—C2	1.4151 (19)	C12—C13	1.487 (2)
C1—Cl1	1.7580 (14)	C13—C14	1.392 (2)
C2—C3	1.366 (2)	C13—C18	1.396 (2)
C2—C11	1.5118 (18)	C14—C15	1.380 (2)
C3—C4	1.4129 (19)	C14—H14	0.93
C3—H3	0.93	C15—C16	1.386 (2)
C4—C10	1.411 (2)	C15—H15	0.93
C4—C5	1.414 (2)	C16—C17	1.379 (2)
C5—C6	1.363 (2)	C16—H16	0.93
C5—H5	0.93	C17—C18	1.385 (2)
C6—C7	1.402 (2)	C17—H17	0.93
C6—H6	0.93	C18—H18	0.93
C7—C8	1.376 (2)	C19—C20	1.4017 (19)
C7—H7	0.93	C19—C24	1.402 (2)
C8—C10	1.427 (2)	C20—C21	1.379 (2)
C8—C9	1.500 (3)	C20—H20	0.93
C9—H9A	0.96	C21—C22	1.385 (2)
C9—H9B	0.96	C21—H21	0.93
C9—H9C	0.96	C22—C23	1.379 (2)
C10—N1	1.3704 (19)	C22—H22	0.93
C11—N3	1.4437 (18)	C23—C24	1.395 (2)
C11—N2	1.4681 (18)	C23—H23	0.93
C11—H11	0.98	C24—N3	1.3753 (17)
C12—N2	1.2924 (17)	N3—H3N	0.86
			
N1—C1—C2	126.42 (13)	C14—C13—C18	118.39 (13)
N1—C1—Cl1	114.91 (11)	C14—C13—C12	120.11 (12)
C2—C1—Cl1	118.67 (10)	C18—C13—C12	121.44 (13)
C3—C2—C1	115.65 (12)	C15—C14—C13	121.05 (13)
C3—C2—C11	120.34 (12)	C15—C14—H14	119.5
C1—C2—C11	123.96 (12)	C13—C14—H14	119.5
C2—C3—C4	121.16 (13)	C14—C15—C16	120.21 (14)
C2—C3—H3	119.4	C14—C15—H15	119.9
C4—C3—H3	119.4	C16—C15—H15	119.9
C10—C4—C3	117.58 (13)	C17—C16—C15	119.22 (14)
C10—C4—C5	119.81 (13)	C17—C16—H16	120.4
C3—C4—C5	122.59 (14)	C15—C16—H16	120.4
C6—C5—C4	120.01 (15)	C16—C17—C18	120.96 (14)
C6—C5—H5	120	C16—C17—H17	119.5
C4—C5—H5	120	C18—C17—H17	119.5
C5—C6—C7	119.94 (14)	C17—C18—C13	120.16 (14)
C5—C6—H6	120	C17—C18—H18	119.9
C7—C6—H6	120	C13—C18—H18	119.9
C8—C7—C6	122.67 (14)	C20—C19—C24	118.54 (13)
C8—C7—H7	118.7	C20—C19—C12	125.02 (13)
C6—C7—H7	118.7	C24—C19—C12	116.21 (12)
C7—C8—C10	117.71 (15)	C21—C20—C19	120.35 (15)
C7—C8—C9	122.46 (14)	C21—C20—H20	119.8
C10—C8—C9	119.83 (15)	C19—C20—H20	119.8
C8—C9—H9A	109.5	C20—C21—C22	120.36 (13)
C8—C9—H9B	109.5	C20—C21—H21	119.8
H9A—C9—H9B	109.5	C22—C21—H21	119.8
C8—C9—H9C	109.5	C23—C22—C21	120.51 (14)
H9A—C9—H9C	109.5	C23—C22—H22	119.7
H9B—C9—H9C	109.5	C21—C22—H22	119.7
N1—C10—C4	121.56 (12)	C22—C23—C24	119.53 (14)
N1—C10—C8	118.58 (14)	C22—C23—H23	120.2
C4—C10—C8	119.85 (14)	C24—C23—H23	120.2
N3—C11—N2	110.52 (11)	N3—C24—C23	122.85 (13)
N3—C11—C2	109.11 (11)	N3—C24—C19	116.63 (12)
N2—C11—C2	110.92 (11)	C23—C24—C19	120.51 (13)
N3—C11—H11	108.7	C1—N1—C10	117.61 (12)
N2—C11—H11	108.7	C12—N2—C11	114.49 (11)
C2—C11—H11	108.7	C24—N3—C11	115.21 (11)
N2—C12—C19	122.44 (13)	C24—N3—H3N	122.4
N2—C12—C13	117.27 (12)	C11—N3—H3N	122.4
C19—C12—C13	120.16 (12)		
			
N1—C1—C2—C3	0.8 (2)	C14—C15—C16—C17	0.4 (2)
Cl1—C1—C2—C3	−178.49 (10)	C15—C16—C17—C18	−0.6 (2)
N1—C1—C2—C11	178.55 (13)	C16—C17—C18—C13	0.7 (2)
Cl1—C1—C2—C11	−0.74 (19)	C14—C13—C18—C17	−0.6 (2)
C1—C2—C3—C4	−0.7 (2)	C12—C13—C18—C17	−177.81 (13)
C11—C2—C3—C4	−178.50 (12)	N2—C12—C19—C20	163.29 (14)
C2—C3—C4—C10	0.1 (2)	C13—C12—C19—C20	−21.0 (2)
C2—C3—C4—C5	−178.31 (13)	N2—C12—C19—C24	−22.4 (2)
C10—C4—C5—C6	−0.1 (2)	C13—C12—C19—C24	153.26 (13)
C3—C4—C5—C6	178.33 (14)	C24—C19—C20—C21	−3.3 (2)
C4—C5—C6—C7	0.9 (2)	C12—C19—C20—C21	170.81 (14)
C5—C6—C7—C8	−1.0 (3)	C19—C20—C21—C22	−0.5 (2)
C6—C7—C8—C10	0.3 (3)	C20—C21—C22—C23	2.9 (2)
C6—C7—C8—C9	−178.86 (18)	C21—C22—C23—C24	−1.3 (2)
C3—C4—C10—N1	0.4 (2)	C22—C23—C24—N3	178.18 (14)
C5—C4—C10—N1	178.87 (13)	C22—C23—C24—C19	−2.6 (2)
C3—C4—C10—C8	−179.12 (14)	C20—C19—C24—N3	−175.84 (13)
C5—C4—C10—C8	−0.6 (2)	C12—C19—C24—N3	9.50 (19)
C7—C8—C10—N1	−179.01 (14)	C20—C19—C24—C23	4.9 (2)
C9—C8—C10—N1	0.2 (2)	C12—C19—C24—C23	−169.73 (13)
C7—C8—C10—C4	0.5 (2)	C2—C1—N1—C10	−0.3 (2)
C9—C8—C10—C4	179.71 (17)	Cl1—C1—N1—C10	178.99 (10)
C3—C2—C11—N3	71.53 (16)	C4—C10—N1—C1	−0.3 (2)
C1—C2—C11—N3	−106.12 (15)	C8—C10—N1—C1	179.22 (14)
C3—C2—C11—N2	−50.45 (17)	C19—C12—N2—C11	−5.44 (19)
C1—C2—C11—N2	131.90 (14)	C13—C12—N2—C11	178.74 (12)
N2—C12—C13—C14	−34.96 (19)	N3—C11—N2—C12	42.97 (16)
C19—C12—C13—C14	149.12 (13)	C2—C11—N2—C12	164.12 (12)
N2—C12—C13—C18	142.19 (14)	C23—C24—N3—C11	−151.84 (13)
C19—C12—C13—C18	−33.73 (19)	C19—C24—N3—C11	28.95 (18)
C18—C13—C14—C15	0.4 (2)	N2—C11—N3—C24	−56.16 (15)
C12—C13—C14—C15	177.67 (13)	C2—C11—N3—C24	−178.38 (12)
C13—C14—C15—C16	−0.4 (2)		

### Hydrogen-bond geometry (Å, º)

**Table:**

*D*—H···*A*	*D*—H	H···*A*	*D*···*A*	*D*—H···*A*
N3—H3*N*···N2^i^	0.86	2.26	3.0998 (18)	165
C11—H11···Cl1	0.98	2.58	3.1166 (16)	115

Symmetry code: (i) −*x*+1, *y*−1/2, −*z*+3/2.
